# Ultrasonographic Fetal Nuchal Translucency Measurements and Cytogenetic Outcomes

**DOI:** 10.1001/jamanetworkopen.2024.3689

**Published:** 2024-03-26

**Authors:** Kara Bellai-Dussault, Shelley D. Dougan, Deshayne B. Fell, Julian Little, Lynn Meng, Nan Okun, Mark C. Walker, Christine M. Armour, Beth K. Potter

**Affiliations:** 1 School of Epidemiology and Public Health, Faculty of Medicine, University of Ottawa, Ottawa, Ontario, Canada; 2 Prenatal Screening Ontario for Better Outcomes Registry & Network Ontario, Ottawa, Canada; 3 Children’s Hospital of Eastern Ontario Research Institute, Ottawa, Canada; 4 DAN Women & Babies Program, Sunnybrook Health Sciences Centre, Toronto, Ontario, Canada; 5 Ottawa Hospital Research Institute, Ottawa, Ontario, Canada; 6 Department of Obstetrics and Gynecology, University of Ottawa, Ottawa, Ontario, Canada; 7 Department of Pediatrics, University of Ottawa, Ottawa, Ontario, Canada

## Abstract

**Importance:**

Ultrasonographic measurement of fetal nuchal translucency is used in prenatal screening for trisomies 21 and 18 and other conditions. A cutoff of 3.5 mm or greater is commonly used to offer follow-up investigations, such as prenatal cell-free DNA (cfDNA) screening or cytogenetic testing. Recent studies showed a possible association with chromosomal anomalies for levels less than 3.5 mm, but extant evidence has limitations.

**Objective:**

To evaluate the association between different nuchal translucency measurements and cytogenetic outcomes on a population level.

**Design, Setting, and Participants:**

This population-based retrospective cohort study used data from the Better Outcomes Registry & Network, the perinatal registry for Ontario, Canada. All singleton pregnancies with an estimated date of delivery from September 1, 2016, to March 31, 2021, were included. Data were analyzed from March 17 to August 14, 2023.

**Exposures:**

Nuchal translucency measurements were identified through multiple-marker screening results.

**Main Outcomes and Measures:**

Chromosomal anomalies were identified through all Ontario laboratory-generated prenatal and postnatal cytogenetic tests. Cytogenetic testing results, supplemented with information from cfDNA screening and clinical examination at birth, were used to identify pregnancies without chromosomal anomalies. Multivariable modified Poisson regression with robust variance estimation and adjustment for gestational age was used to compare cytogenetic outcomes for pregnancies with varying nuchal translucency measurement categories and a reference group with nuchal translucency less than 2.0 mm.

**Results:**

Of 414 268 pregnancies included in the study (mean [SD] maternal age at estimated delivery date, 31.5 [4.7] years), 359 807 (86.9%) had a nuchal translucency less than 2.0 mm; the prevalence of chromosomal anomalies in this group was 0.5%. An increased risk of chromosomal anomalies was associated with increasing nuchal translucency measurements, with an adjusted risk ratio (ARR) of 20.33 (95% CI, 17.58-23.52) and adjusted risk difference (ARD) of 9.94% (95% CI, 8.49%-11.39%) for pregnancies with measurements of 3.0 to less than 3.5 mm. The ARR was 4.97 (95% CI, 3.45-7.17) and the ARD was 1.40% (95% CI, 0.77%-2.04%) when restricted to chromosomal anomalies beyond the commonly screened aneuploidies (excluding trisomies 21, 18, and 13 and sex chromosome aneuploidies).

**Conclusions and Relevance:**

In this cohort study of 414 268 singleton pregnancies, those with nuchal translucency measurements less than 2.0 mm were at the lowest risk of chromosomal anomalies. Risk increased with increasing measurements, including measurements less than 3.5 mm and anomalies not routinely screened by many prenatal genetic screening programs.

## Key Points

**Question:**

Is there an association between nuchal translucency measurements less than 3.5 mm and chromosomal anomalies?

**Findings:**

In this population-based cohort study including 414 268 singleton pregnancies in Ontario, Canada, a significantly increased risk of chromosomal anomalies was associated with each increasing level of nuchal translucency measurement, compared with a reference group of pregnancies with nuchal translucencies less than 2.0 mm.

**Meaning:**

The findings of this cohort study suggest that pregnancies with nuchal translucency measurements greater than 2.0 mm are at increased risk of chromosomal anomalies, indicating that the widely used threshold of 3.5 mm may need to be reexamined.

## Teaser

This cohort study evaluates the association between all levels of nuchal translucency measurements and cytogenetic outcomes among singleton pregnancies in Ontario, Canada, identified through a population-based provincial registry.

## Introduction

Since the 1990s, ultrasonographic measurement of nuchal translucency—a collection of fluid behind the fetal neck^1^—has been included in prenatal genetic screening offered to pregnant individuals to identify trisomies 21 and 18, typically as a part of multiple-marker screening.^2,3^ In addition to these aneuploidies, increased fetal nuchal translucency is associated with other chromosomal anomalies, single gene conditions, and structural defects.^4^ The current practice in many jurisdictions, including Ontario, Canada, is to offer follow-up investigations when nuchal translucency is greater than or equal to 3.5 mm, which theoretically corresponds to the 99th percentile for all gestational ages.^5,6,7,8,9^ These follow-up investigations may include prenatal cell-free DNA (cfDNA) screening and/or confirmatory diagnostic testing through cytogenetic analysis.

Many studies of the association between nuchal translucency measurement and chromosomal anomalies beyond trisomies 21 and 18 have been beset by methodological limitations such as selection bias, for example, by focusing on narrow or high-risk populations (eg, based at tertiary institutions)^10,11,12,13,14^ or including only pregnant individuals who elected to have a prenatal diagnosis.^15,16,17,18^ Studies have also been limited by failing to include a low nuchal translucency reference group, including a historical reference group only, having a small sample size, or including only prenatal cytogenetic testing results.^19,20,21,22,23^ A small number of these studies^19,20,22^ have provided preliminary evidence that pregnancies with nuchal translucency measurements that are elevated but still lower than 3.5 mm could also be at increased risk of clinically significant chromosomal anomalies. These findings require confirmation using robust methodological approaches in large, unselected samples with comprehensive follow-up to adequately assess the risk of chromosomal anomalies across the entire range of nuchal translucency measurements. In this study, we aimed to evaluate the association between all levels of nuchal translucency measurements and cytogenetic outcomes among pregnancies in Ontario, Canada, identified through a population-based provincial registry.

## Methods

This study received approval from the research ethics boards of the Children’s Hospital of Eastern Ontario and the University of Ottawa. The requirement of informed consent was waived owing to the use of deidentified patient data. All cell counts of less than 6 were suppressed to comply with the privacy requirements of the registry. The study followed the Reporting of Studies Conducted Using Observational Routinely–Collected Health Data (RECORD) reporting guideline.

### Data Source

Better Outcomes Registry & Network (BORN) Ontario is a prescribed perinatal registry that collects data directly from all multiple-marker screening, cfDNA screening, and cytogenetics laboratories in Ontario.^24^ Pregnancy and birth outcomes are also captured, including information on the clinical birth examination and linkage to discharge data from all hospitals through the discharge abstract database of the Canadian Institute for Health Information (CIHI). This enabled comprehensive ascertainment of pregnancy outcomes. Additional details on data sources are provided in eTable 1 in Supplement 1.

### Setting and Study Population

Ontario offers a publicly funded screening program in which all pregnant individuals have access to multiple-marker screening in the first trimester, most often including a nuchal translucency measurement.^25^ Cell-free DNA screening or cytogenetic testing is offered if the screen result is positive or as a first-tier screen if specific eligibility criteria are met.^26^ Individuals may also self-pay for cfDNA screening.^26^

Nuchal translucencies are measured at crown-rump lengths of 45 to 84 mm by sonographers registered in Ontario’s Nuchal Translucency Quality Assurance program.^27^ This study included all singleton pregnancies in Ontario with a valid multiple-marker screening test including a nuchal translucency and with an estimated date of delivery (EDD) from September 1, 2016, to March 31, 2021 (eFigure 1 in Supplement 1). While ethnicity is recorded in the BORN database, we did not incorporate this information into our analysis as it was not expected to influence the associations studied.

### Study Exposure

Nuchal translucency measurements for all pregnancies were identified from multiple-marker screening results. The reference group was defined as pregnancies with a measurement less than 2.0 mm, compared with pregnancies with the following categories of nuchal translucency measurements: 2.0 to less than 2.5 mm, 2.5 to less than 3.0 mm, 3.0 to less than 3.5 mm, 3.5 to less than 5.0 mm, 5.0 to less than 6.5 mm, and 6.5 mm or greater.

### Study Outcome

Pregnancies with chromosomal anomalies were identified through cytogenetic testing results submitted by all Ontario cytogenetic laboratories to the BORN registry. The primary outcome was defined as any chromosomal anomaly identified on cytogenetic testing, during pregnancy or postnatally, including microarray analysis. As secondary outcomes, we stratified chromosomal anomalies by whether or not the condition is routinely tested through cfDNA screening in Ontario (trisomies 21, 18, and 13 and sex chromosome aneuploidies).

Because only a small number of pregnancies have cytogenetic investigations, we supplemented our outcome data with information from other sources (pregnancies with and without cytogenetic testing are described in eTable 12 in Supplement 1). Specifically, to identify pregnancies without chromosomal anomalies, we first used cytogenetic testing results if performed. If no cytogenetic testing results were available, we used cfDNA screening results, if performed; although a low-risk cfDNA screening result cannot be used clinically to exclude these conditions (trisomies 21, 18, and 13 and sex chromosome aneuploidies), for the purposes of this research, it was considered a reasonable proxy given its negative predictive value of greater than 99.9%.^28^ Finally, for pregnancies with no cytogenetic testing and no cfDNA screening, we used results from the clinical examination at birth to exclude conditions typically clinically diagnosable at birth, relying on both BORN and CIHI data (eTable 2 in Supplement 1).

### Statistical Analysis

Data were analyzed from March 17 to August 14, 2023. The study population was described using means (SDs) for continuous variables and frequencies and proportions for categorical variables. We used multivariable modified Poisson regression models with robust variance estimation and adjustment for gestational age at screening to compare the risk of chromosomal anomalies across pregnancies with varying categories of nuchal translucency measurements with the reference category (<2.0 mm).^29^ This model also allowed us to account for clustering for individuals with more than 1 pregnancy within the study period.^30^ Gestational age at screening was identified a priori as a potential confounder through directed acyclic graphs, as nuchal translucency measurements are on a continuum and will change with gestational age (eFigure 2 in Supplement 1). A post hoc analysis with additional adjustment for age of the pregnant individual was also performed. Adjusted risk ratios (ARRs) and risk differences (ARDs) were reported with 95% CIs.

We conducted the following sensitivity analyses to evaluate the potential impact of incomplete or inaccurate ascertainment of the exposure and outcome and to address losses to follow-up. All analyses were performed using SAS, version 9.4 (SAS Institute Inc), and 2-tailed *P* < .05 was considered statistically significant.

#### Exposure Measurement Source

When a very high nuchal translucency measurement or cystic hygroma is identified, some pregnant individuals may not complete the multiple-marker screening process and, thus, may not be ascertained by the laboratories. We therefore performed a sensitivity analysis identifying pregnancies with increased nuchal translucency measurements through other sources available within the registry, including data obtained from consultations with genetics or maternal fetal medicine clinics and from documented clinical indications for testing obtained from cytogenetic laboratories (eTable 3 in Supplement 1).

#### Exposure Definition

Some studies rely on percentiles of the nuchal translucency measurement rather than absolute values. Therefore, we categorized nuchal translucency measurements as less than 90th, 90th to less than 95th, 95th to less than 99th, and 99th percentile or greater^10,21^ in an additional sensitivity analysis (eTable 4 in Supplement 1).

#### Losses to Follow-Up

For some pregnancies, no outcome was recorded. These may reflect pregnancy losses or terminations in the absence of follow-up cfDNA screening or cytogenetic testing or pregnant individuals who had multiple-marker screening in Ontario but subsequently received care outside the province. Because of the unclear nature of the outcome for these pregnancies, we performed a sensitivity analysis in which we randomly classified the losses to follow-up to having twice the prevalence of chromosomal anomalies compared with pregnancies in the same category of nuchal translucency measurement for which an outcome was recorded or half the prevalence (eTable 5 in Supplement 1). A further sensitivity analysis included all pregnancies with varying assumptions of risk of chromosomal anomalies for those lost to follow-up based on the pregnancy outcome (eTable 6 in Supplement 1).

#### Outcome

Complete cytogenetic data for microarray testing was only available since January 2018. Therefore, we conducted an analysis restricted to pregnancies with EDD from September 1, 2018, to March 31, 2021 (eTable 7 in Supplement 1).

#### Time Period

An additional sensitivity analysis excluded pregnancies with an EDD from April 1, 2020, to March 31, 2021. This exclusion accounted for potential effects of the COVID-19 pandemic on prenatal care practices (eTable 8 in Supplement 1).

## Results

From 643 146 singleton pregnancies in Ontario during the study period, 414 268 were eligible for the analysis (mean [SD] maternal age at EDD, 31.5 [4.7] years). Of these, 359 807 pregnancies (86.9%) had a nuchal translucency measurement less than 2.0 mm; 43 219 (10.4%), from 2.0 to less than 2.5 mm; 7474 (1.8%), from 2.5 to less than 3.0 mm; 1789 (0.4%), from 3.0 to less than 3.5 mm; 1088 (0.3%), from 3.5 to less than 5.0 mm; 404 (0.1%), from 5.0 to less than 6.5 mm; and 487 (0.1%), 6.5 mm or greater (Table 1). The mean (SD) maternal age at EDD increased across nuchal translucency categories, from 31.5 (4.7) years for pregnant individuals with nuchal translucency measurements less than 2.0 mm to 33.3 (5.4) years for those with measurements 6.5 mm or greater. We excluded 225 264 pregnancies without a valid multiple-marker screening test including a nuchal translucency measurement, 158 with no screening result report issued, and 3456 where the measurement was performed outside the gestational age range corresponding to the crown-rump length of 45 to 84 mm.

**Table 1.** Characteristics of Study Population by Nuchal Translucency Measurement

Characteristic	All pregnancies with nuchal translucency measurement (N = 414 268)	Nuchal translucency measurement, mm						
		<2.0 (n = 359 807)	2.0 to <2.5 (n = 43 219)	2.5 to <3.0 (n = 7474)	3.0 to <3.5 (n = 1789)	3.5 to <5.0 (n = 1088)	5.0 to <6.5 (n = 404)	≥6.5 (n = 487)
Maternal age at EDD, mean (SD), y	31.5 (4.7)	31.5 (4.7)	31.6 (4.8)	31.9 (4.8)	32.2 (4.8)	32.8 (5.0)	33.0 (5.5)	33.3 (5.4)
Gestational age at screening, mean (SD), d	87.8 (3.3)	87.5 (3.3)	90.0 (2.6)	90.0 (3.0)	88.9 (3.4)	87.3 (3.6)	86.4 (3.6)	86.9 (3.1)
Crown rump length, mean (SD), mm	62.5 (8.3)	61.7 (8.1)	68.5 (7.5)	68.8 (8.2)	65.6 (8.9)	61.6 (9.0)	59.2 (8.7)	60.3 (7.5)
No. missing	199	134	53	10	<6	0	0	<6
Maternal weight, mean (SD), kg	68.0 (17.0)	67.9 (16.9)	68.5 (17.4)	68.3 (17.3)	68.1 (17.0)	67.2 (16.6)	68.8 (17.8)	67.0 (14.0)
No. missing	18 316	14 783	2009	448	209^a^	297^a^	227^a^	343^a^
Parity, No. (%)								
Nulliparous	183 587 (46.2)	161 994 (46.8)	17 689 (42.7)	2847 (40.3)	595 (37.5)	314 (39.4)	81 (46.0)	67 (45.6)
Primiparous	143 190 (36.0)	123 661 (35.7)	15 760 (38.0)	2739 (38.7)	614 (38.7)	320 (40.2)	56 (31.8)	40 (27.2)
Multiparous	70 967 (17.8)	60 848 (17.6)	8012 (19.3)	1487 (21.0)	378 (23.8)	163 (20.5)	39 (22.2)	40 (27.2)
No. missing	16 524	13 304	1758	401	202^a^	291^a^	228^a^	340^a^
Type of conception, No. (%)								
Spontaneous conception	374 873 (96.0)	326 450 (96.0)	38 712 (95.9)	6685 (95.9)	1568 (96.1)	876 (96.4)	275 (96.8)	307 (97.5)
IVF	12 025 (3.1)	10 429 (3.1)	1289 (3.2)	220 (3.2)	49 (3.0)	22 (2.4)	8 (2.8)	8 (2.5)
Other ART	3659 (0.9)	3188 (0.9)	378 (0.9)	66 (0.9)	15 (0.9)	11 (1.2)	<6 (NA)	0
No. missing	23 711	19 740	2840	503	157	179^a^	120^a^	172^a^

Abbreviations: ART, assisted reproductive technology; EDD, estimated date of delivery; IVF, in vitro fertilization; NA, not applicable.

^a^ Missing data for more than 10.0% of the pregnancies.

**Figure 1.**
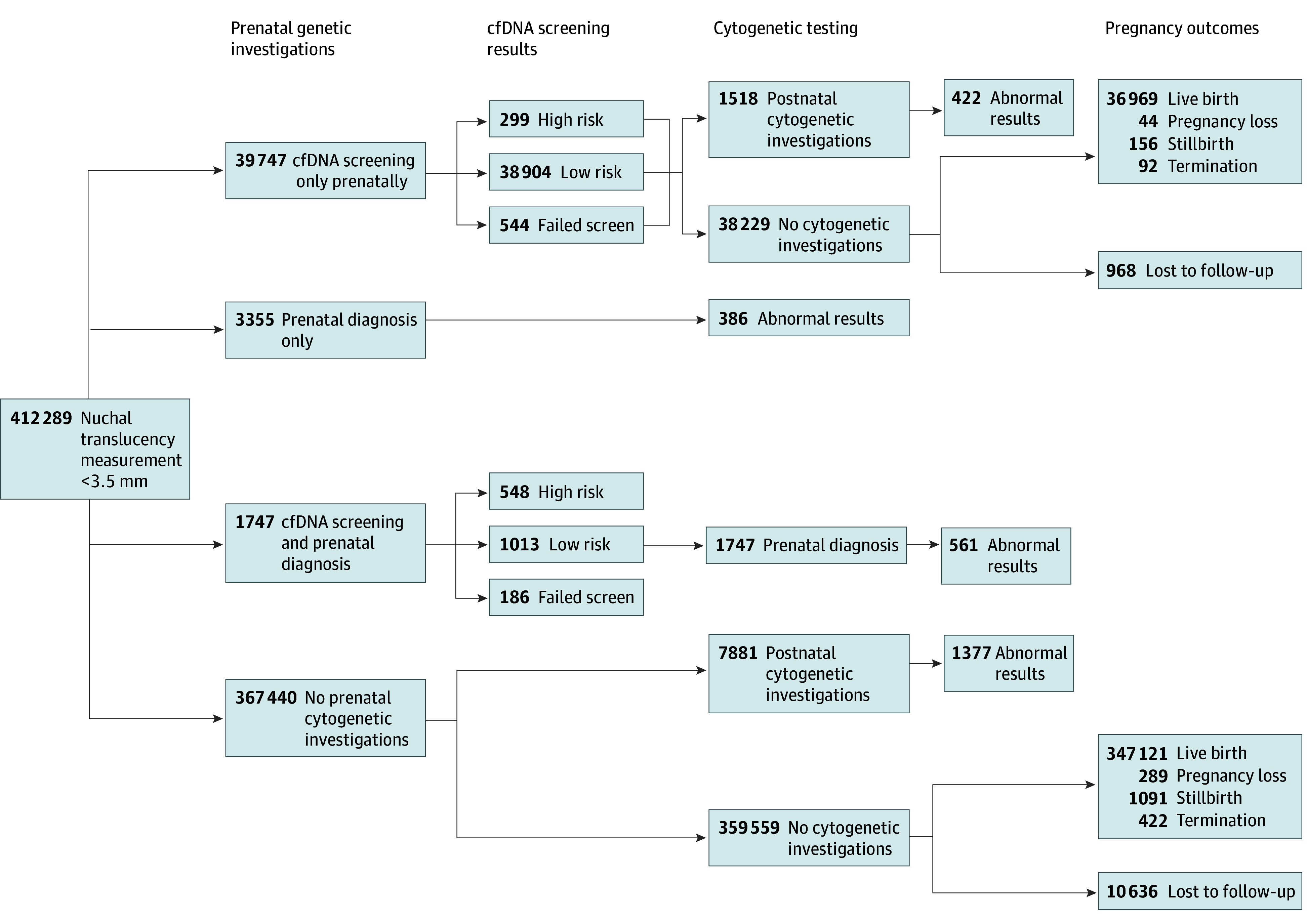
Investigations Following an Ultrasonographic Nuchal Translucency Measurement Less Than 3.5 mm Under Current Practice cfDNA indicates cell-free DNA.

**Figure 2.**
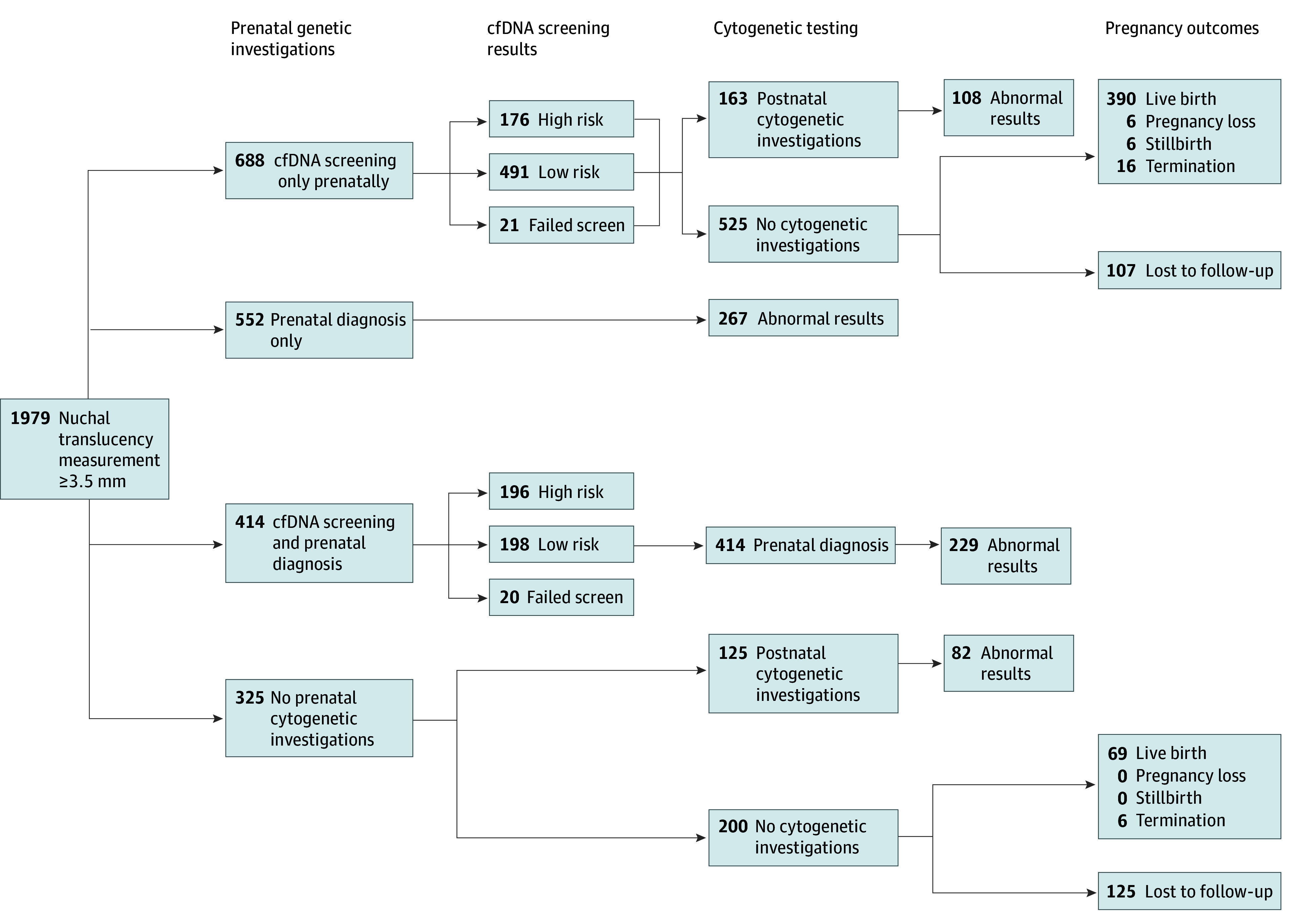
Investigations Following an Ultrasonographic Nuchal Translucency Measurement 3.5 mm or Greater Under Current Practice cfDNA indicates cell-free DNA.

Figure 1 and Figure 2 describe the uptake of follow-up investigations (cfDNA screening, cytogenetic testing) and pregnancy outcomes among pregnant individuals with nuchal translucency measurements less than 3.5 mm and 3.5 mm or greater, respectively. Of pregnancies with a nuchal translucency measurement 3.5 mm or greater, 1654 (83.6%) underwent follow-up investigations prenatally, compared with 44 849 (10.9%) of pregnancies with a measurement less than 3.5 mm. Among those with nuchal translucencies 3.5 mm or greater, 414 (20.9%) had both cfDNA screening and prenatal diagnosis with cytogenetic testing, compared with 1747 (0.4%) among pregnancies with a measurement less than 3.5 mm. Of chromosomal anomalies identified in pregnancies with measurements of 3.5 mm or greater, 496 (72.5%) were identified prenatally, compared with 947 (35.3%) for measurements less than 3.5 mm (eTable 9 in Supplement 1).

Among pregnancies with cytogenetic testing results (n = 15 755), the proportion with chromosomal anomalies increased across nuchal translucency measurement categories, from 1913 pregnancies (16.6%) with nuchal translucency less than 2.0 mm to 256 pregnancies (70.1%) with a measurement 6.5 mm or greater (Table 2). To ascertain potential chromosomal anomalies in pregnancies that did not receive cytogenetic testing, 38 041 (98.2%) received a low-risk cfDNA screening result (Table 2). Next, among pregnancies with no cfDNA screening performed, we identified 331 638 documented live births with no notable clinical findings reported (Table 2). These results were used to estimate the proportion of pregnancies in the study with chromosomal anomalies, increasing from 1913 (0.5%) in pregnancies with nuchal translucency less than 2.0 mm to 256 (52.6%) in pregnancies with nuchal translucency 6.5 mm or greater (Table 2).

**Table 2.** Chromosomal and Pregnancy Outcomes by Nuchal Translucency Measurement^a^

Outcome	All pregnancies	Nuchal translucency measurement, mm						
		<2.0	2.0 to <2.5	2.5 to <3.0	3.0 to <3.5	3.5 to <5.0	5.0 to <6.5	≥6.5
No. of cytogenetic testing results	15 755	11 552	1875	653	421	602	287	365
Unknown result	217 (1.4)	180 (1.6)	22 (1.2)	<6 (NA)	<6 (NA)	<6 (NA)	<6 (NA)	<6 (NA)
No chromosomal anomaly identified	12 106 (76.8)	9459 (81.9)	1397 (74.5)	450 (68.9)	241 (57.2)	341 (56.6)	112 (39.0)	106 (29.0)
Chromosomal anomaly	3432 (21.8)	1913 (16.6)	456 (24.3)	198 (30.3)	179 (42.5)	256 (42.5)	174 (60.6)	256 (70.1)
Identifiable on cfDNA screening^b^^,^^c^	1690 (49.2)	543 (28.4)	241 (52.9)	154 (77.8)	149 (83.2)	215 (84.0)	153 (87.9)	235 (91.8)
Not identifiable on cfDNA screening^b^^,^^d^	1742 (50.8)	1370 (71.6)	215 (47.1)	44 (22.2)	30 (16.8)	41 (16.0)	21 (12.1)	21 (8.2)
No. of cfDNA screening results for pregnancies without cytogenetic testing results	38 754	30 551	5310	1701	667	398	69	58
Unknown or uninformative result	495 (1.3)	404 (1.3)	58 (1.1)	14 (0.8)	<6 (NA)	8 (2.0)	<6 (NA)	<6 (NA)
Low-risk result	38 041 (98.2)	30 071 (98.4)	5234 (98.6)	1674 (98.4)	645 (96.7)	354 (88.9)	44 (63.8)	19 (32.8)
High-risk result	218 (0.6)	76 (0.2)	18 (0.3)	13 (0.8)	18 (2.7)	36 (9.0)	21 (30.4)	36 (62.1)
No. of pregnancy outcomes with neither cytogenetic testing nor cfDNA screening results	359 759	317 704	36 034	5120	701	88	48	64
Lost to follow-up	10 761 (3.0)	9281 (2.9)	1132 (3.1)	181 (3.5)	42 (6.0)	38 (43.2)	39 (81.3)	48 (75.0)
Live birth	347 190 (96.5)	306 838 (96.6)	34 727 (96.4)	4901 (95.7)	655 (93.4)	49 (55.7)	7 (14.6)	13 (20.3)
Clinical findings at birth^e^	15 552 (4.5)	13 919 (4.5)	1391 (4.0)	203 (4.1)	33 (5.0)	<6 (NA)	<6 (NA)	<6 (NA)
Pregnancy loss, termination, or stillbirth	1808 (0.5)	1585 (0.5)	175 (0.5)	38 (0.7)	<6 (NA)	<6 (NA)	<6 (NA)	<6 (NA)
No. of pregnancies with chromosomal anomalies	414 268	359 807	43 219	7474	1789	1088	404	487
No chromosomal anomaly identified^f^	382 478 (92.3)	332 945 (92.5)	40 046 (92.7)	6846 (91.6)	1522 (85.1)	771 (70.9)	179 (44.3)	169 (34.7)
Chromosomal anomaly^g^	3432 (0.8)	1913 (0.5)	456 (1.1)	198 (2.6)	179 (10.0)	256 (23.5)	174 (43.1)	256 (52.6)
Excluded	28 358 (6.8)	24 949 (6.9)	2717 (6.3)	430 (5.8)	88 (4.9)	61 (5.6)	51 (12.6)	62 (12.7)
Lost to follow-up	10 761 (2.6)	9281 (2.6)	1132 (2.6)	181 (2.4)	42 (2.3)	38 (3.5)	39 (9.7)	48 (9.9)
Excluded for other reason^h^	17 597 (4.2)	15 668 (4.4)	1585 (3.7)	249 (3.3)	46 (2.6)	23 (2.1)	12 (3.0)	14 (2.9)

Abbreviations: cfDNA, cell-free DNA; NA, not applicable.

^a^ Unless otherwise indicated, data are expressed as No. (%) of patients.

^b^ Includes those with choromosomal anomaly identified.

^c^ Includes trisomies 21, 18, and 13 and sex chromosome aneuploidies.

^d^ Includes all other chromosomal anomalies, including other autosomal aneuploidies, mosaic aneuploidies, copy number variants. A detailed list of all chromosomal anomalies is available in eTable 2 in Supplement 1.

^e^ Includes any congenital structural anomaly identified among live births.

^f^ Indicates normal cytogenetic results or no cytogenetic results but low risk cfDNA results or no follow-up testing results but documented live birth with no clinical findings on examination.

^g^ Based on cytogenetic testing results.

^h^ Includes pregnancy loss, stillbirth, termination, live birth with clinical findings, or a high-risk cfDNA screening result.

The risk of chromosomal anomalies increased with increasing nuchal translucency measurements (Table 3). The risk was markedly increased in pregnancies with nuchal translucency measurements from 3.0 to less than 3.5 mm relative to less than 2.0 mm (ARD, 9.94% [95% CI, 8.49%-11.39%]; ARR, 20.33 [95% CI, 17.58-23.52]).

**Table 3.** Chromosomal Anomalies by Nuchal Translucency Measurement^a^

Nuchal translucency measurement, mm	Main analysis				Subgroup analysis, adjusted model^b^			
	Crude model		Adjusted model^b^		Conditions identifiable on cfDNA screening^c^		Conditions not identifiable on cfDNA screening^d^	
	RD (95% CI), %	RR (95% CI)	RD (95% CI), %	RR (95% CI)	RD (95% CI), %	RR (95% CI)	RD (95% CI), %	RR (95% CI)
<2.0	1 [Reference]	1 [Reference]	1 [Reference]	1 [Reference]	1 [Reference]	1 [Reference]	1 [Reference]	1 [Reference]
2.0 to <2.5	0.55 (0.45-0.66)	1.97 (1.78-2.18)	0.61 (0.51-0.71)	2.39 (2.14-2.66)	0.45 (0.37-0.52)	4.21 (3.62-4.91)	0.16 (0.08-0.23)	1.48 (1.27-1.73)
2.5 to <3.0	2.24 (1.85-2.63)	4.92 (4.26-5.69)	2.26 (1.88-2.63)	5.93 (5.11-6.88)	2.01 (1.67-2.34)	14.76 (12.33-17.65)	0.27 (0.08-0.46)	1.82 (1.34-2.47)
3.0 to <3.5	9.95 (8.49-11.41)	18.42 (15.92-21.31)	9.94 (8.49-11.39)	20.33 (17.58-23.52)	8.62 (7.27-9.96)	52.15 (43.98-61.84)	1.40 (0.77-2.04)	4.97 (3.45-7.17)
3.5 to <5.0	24.36 (21.71-27.00)	43.63 (38.88-48.96)	24.31 (21.67-26.96)	42.94 (38.28-48.16)	21.10 (18.60-23.61)	107.31 (93.20-123.56)	3.50 (2.27-4.72)	10.15 (7.37-13.98)
5.0 to <6.5	48.72 (43.51-53.94)	86.28 (76.91-96.80)	48.68 (43.46-53.89)	80.85 (71.91-90.90)	43.97 (38.79-49.15)	208.01 (179.77-240.69)	5.15 (2.66-7.64)	14.17 (9.02-22.25)
≥6.5	59.66 (54.99-64.33)	105.44 (96.40-115.32)	59.64 (54.97-64.31)	101.88 (92.79-111.88)	55.79 (51.06-60.53)	276.14 (244.84-311.44)	4.30 (2.18-6.41)	12.11 (7.69-19.07)

Abbreviations: cfDNA, cell-free DNA; RD, risk difference; RR, risk ratio.

^a^ Outcome determined by cytogenetic testing. Not having the outcome is determined by normal cytogenetic testing result if performed, normal cfDNA screen result if condition was tested, and by live birth without clinical findings if condition can be clinically diagnosed.

^b^ Adjusted for gestational age at screening.

^c^ Includes trisomies 21, 18, and 13 and sex chromosome aneuploidies.

^d^ Includes all other chromosomal anomalies.

The proportion of pregnancies excluded from the primary analysis due to an unknown outcome was associated with nuchal translucency category, from 9281 (2.6%) in the group with measurements less than 2.0 mm to 48 (9.9%) in the group with measurements 6.5 mm or greater (Table 2). We therefore conducted a sensitivity analysis randomly classifying the pregnancies lost to follow-up to have twice the prevalence of chromosomal anomalies compared with those for which an outcome was recorded within the given nuchal translucency measurement category, and results were mildly accentuated. The analysis assuming half the prevalence showed mildly attenuated results (eTable 5 in Supplement 1).

Table 3 further categorizes chromosomal anomalies into a group of conditions routinely screened by cfDNA screening (trisomies 21, 18, and 13 and sex chromosome aneuploidies) and a group of conditions beyond the cfDNA screening options consistently available in Ontario (other autosomal aneuploidies, triploidy, mosaic autosomal and sex chromosome aneuploidies, and copy number variants). The risk of chromosomal anomalies routinely screened by cfDNA screening increased with increasing nuchal translucency measurements: for the nuchal translucency category of 3.0 to less than 3.5 mm relative to less than 2.0 mm, the ARD was 8.62% (95% CI, 7.27%-9.96%) and the ARR was 52.15 (95% CI, 43.98-61.84). The risk also increased but with weaker magnitude for the subgroup with other chromosomal anomalies (detailed in eTable 11 in Supplement 1): for nuchal translucency category of 3.0 to less than 3.5 mm relative to less than 2.0 mm, the ARD was 1.40% (95% CI, 0.77%-2.04%) and the ARR was 4.97 (95% CI, 3.45-7.17).

All additional sensitivity analyses showed comparable findings to the primary analysis, including analyses incorporating pregnancies with nuchal translucency measurements identified by sources other than multiple-marker screening (eTable 3 in Supplement 1); analyses restricted to a timeline with complete capture of microarray data, with an EDD from September 1, 2018, to March 31, 2021 (eTable 7 in Supplement 1); analyses excluding pregnancies with an EDD from April 1, 2020, to March 31, 2021, to assess potential effects of the COVID-19 pandemic (eTable 8 in Supplement 1); and post hoc analyses additionally adjusting for age of the pregnant individual (eTable 10 in Supplement 1). Finally, for the pregnancies included in our study, the 99th percentile for nuchal translucency measurement was 2.8 mm, the 95th percentile was 2.2 mm, and the 90th percentile was 2.0 mm. When defining the exposure by nuchal translucency percentile, pregnancies with a measurement greater than the 99th percentile had a risk of any chromosomal anomaly 34.9 times greater than pregnancies with a measurement less than the 90th percentile (eTable 4 in Supplement 1).

## Discussion

This population-based cohort study leveraged linked multiple-marker screening, cytogenetic testing, cfDNA screening, and birth registry data capturing pregnancy outcomes and findings from the clinical examination at birth to quantify the association of increased risk of chromosomal anomalies with increasing nuchal translucency measurement. We found a strongly increased risk of chromosomal anomalies with increased nuchal translucency relative to values less than 2.0 mm, particularly for measurements of 3.0 mm or higher. The findings were consistent through several sensitivity analyses.

To our knowledge, this is the first population-based study assessing the risk of chromosomal anomalies across all levels of nuchal translucency measurements and incorporating information from antenatal as well as postnatal cytogenetic testing, cfDNA screening, pregnancy outcomes, and newborn clinical examinations. Indeed, most studies on this topic have focused on high-risk settings or have included only prenatal cytogenetic testing and, therefore, only represent a small proportion of pregnant individuals undergoing nuchal translucency ultrasonography.^11,13,14,16,17,18,21,31^ The concern is that pregnant individuals who opted for cytogenetic testing in these studies may have had a higher risk of chromosomal anomalies, as additional findings beyond nuchal translucency measurement may have led them to have prenatal diagnostic testing; excluding pregnancies at lower risk for which outcomes are not available would, therefore, tend to overestimate the risk. This is particularly important when investigating nuchal translucency measurement values that would not independently trigger an offer of a follow-up investigation. As expected, we observed a substantial difference in the proportion of pregnant individuals who elected to have follow-up investigations prenatally in the group with nuchal translucency measurements less than 3.5 mm (10.9%) compared with the group with measurements of at least 3.5 mm (83.6%), illustrating the importance of including outcomes beyond cytogenetic results from prenatal diagnosis.

Our finding that nuchal translucency values below 3.5 mm, particularly those from 3.0 to less than 3.5 mm, are associated with chromosomal anomalies relative to values less than 2.0 mm, has important implications for prenatal genetic screening and counselling. Beyond the common aneuploidies (trisomies 21, 13, and 18 and sex chromosome aneuploidies) routinely identified by cfDNA screening in Ontario and included in many screening programs internationally,^32^ we report that increased nuchal translucency measurements also yield an increased risk for other chromosome anomalies, although weaker. This has important policy implications given that in some jurisdictions, prenatal cfDNA screening is offered following the identification of an increased nuchal translucency measurement, whereas in others, cfDNA screening is part of first-tier prenatal screening, with or without accompanying nuchal translucency measurement. Screening programs should consider the value of nuchal translucency measurements when making decisions about whether to replace such measurements with cfDNA screening alone and may wish to reexamine the threshold of nuchal translucency at which diagnostic investigations are offered.^10,32^ Further research, including economic modeling to estimate benefits and costs, is needed to inform the best options for policy and practice.^10,32^

The results of this study also have implications for the quality assurance of nuchal translucency: the 99th percentile for nuchal translucency measurement was well below the expected 3.5 mm, implying that using a cutoff of 3.5 mm to offer follow-up investigations may not be sufficient. While the increased risk of chromosomal anomalies in pregnancies with nuchal translucencies less than 3.5 mm could be partly due to chronic undermeasurement, it is unlikely that this would be the only factor. Indeed, some jurisdictions have adopted thresholds to offer follow-up investigations much lower than the 99th percentile (eg, 95th percentile in Finland, the Netherlands, Germany, and Switzerland).^33^ These findings also highlight the importance of a robust quality assurance program to support nuchal translucency measurement, as chronic undermeasurement can reduce the screening sensitivity.^34,35,36,37,38,39^

### Limitations

An inherent limitation of this study is that cytogenetic outcomes were not available for all pregnancies, as cytogenetic testing is only offered under specific clinical indications. We therefore included additional information from cfDNA screening and pregnancy outcomes recorded in the birth registry to maximize ascertainment of chromosomal anomalies. Moreover, sensitivity analyses with varying assumptions about outcome ascertainment yielded the same conclusions.

Additionally, some chromosomal anomalies may not have clinically significant features at birth, prompting postnatal cytogenetic investigations, such that an infant could possibly be misclassified as not having a chromosomal anomaly. Because of the difference in ascertainment of pregnancies with nuchal translucencies less than 3.5 mm and 3.5 mm or greater, it is possible that we overestimated the risk of chromosomal anomalies in pregnancies with measurements of 3.5 mm or greater. For this reason, our findings allow us to draw conclusions on the association between nuchal translucency measurements and chromosomal anomalies that have clear features at birth, whereas more careful consideration is needed for chromosomal anomalies for which a clear phenotype is not expected at birth. Additionally, single-gene conditions associated with increased nuchal translucency measurements such as RASopathies are not captured in the registry and were therefore not included.^40^ Although there is evidence that some factors may influence the choice to have prenatal genetic screening (eg, maternal age, rural residence),^25,41^ there is no reason to expect the association between the nuchal translucency measurement and chromosomal anomalies to differ in this population excluded from our study.

## Conclusions

The findings of this population-based cohort study suggest that increased nuchal translucency measurements were associated with increased risk of chromosomal anomalies, even at values below the currently used standard threshold of 3.5 mm. These findings have important policy implications for setting the threshold nuchal translucency value such that further investigations may be offered to pregnant individuals and, in turn, contribute to determining the best approach to offering high-quality prenatal screening to pregnant individuals in Ontario and around the world.

## Notes

Supplement 1. **eTable 1.** Description of Data Sources **eTable 2.** Method of Ascertainment of Outcome **eFigure 1.** Inclusion Flow **eFigure 2.** Directed Acyclic Graph for Association Between Nuchal Translucency Measurement and Chromosomal Anomalies **eTable 3.** Regression Models Including Pregnancies Identified Through Other Data Sources Beyond Multiple-Marker Screening **eTable 4.** Regression Model With Varying Definition of Exposure by Nuchal Translucency Percentile **eTable 5.** Sensitivity Analysis for Losses to Follow-Up **eTable 6.** Regression Models Including All Pregnancies With Nuchal Translucency Measurements **eTable 7.** Regression Models Including Only Pregnancies With an Estimated Date of Delivery From September 1, 2018, to March 31, 2021 **eTable 8.** Regression Models Excluding Pregnancies With an Estimated Date of Delivery From April 1, 2020, to March 31, 2021 **eTable 9.** Time of Cytogenetic Testing of Chromosomal Anomalies Identified by Nuchal Translucency Measurement **eTable 10.** Post Hoc Analysis Including Additional Adjustment for Age of the Pregnant Individual at Estimated Date of Delivery **eTable 11.** Chromosomal and Pregnancy Outcomes by Nuchal Translucency Measurement: Detailed Results **eTable 12.** Characteristics of Pregnancies With and Without Cytogenetic Testing

Supplement 2.
Data Sharing Statement
