# Supplementary material for: Ultrasonographic Fetal Nuchal Translucency Measurements and Cytogenetic Outcomes
Source: JAMA Netw Open. 2024 Mar 26;7(3):e243689. doi: 10.1001/jamanetworkopen.2024.3689 (PMC10966411; doi:10.1001/jamanetworkopen.2024.3689)
Supplement: Supplement 1. — eTable 1. Description of Data Sources eTable 2. Method of Ascertainment of Outcome eFigure 1. Inclusion Flow eFigure 2. Directed Acyclic Graph for Association Between Nuchal Translucency Measurement and Chromosomal Anomalies eTable 3. Regression Models Including Pregnancies Identified Through Other Data Sources Beyond Multiple-Marker Screening eTable 4. Regression Model With Varying Definition of Exposure by Nuchal Translucency Percentile eTable 5. Sensitivity Analysis for Losses to Follow-Up eTable 6. Regression Models Including All Pregnancies With Nuchal Translucency Measurements eTable 7. Regression Models Including Only Pregnancies With an Estimated Date of Delivery From September 1, 2018, to March 31, 2021 eTable 8. Regression Models Excluding Pregnancies With an Estimated Date of Delivery From April 1, 2020, to March 31, 2021 eTable 9. Time of Cytogenetic Testing of Chromosomal Anomalies Identified by Nuchal Translucency Measurement eTable 10. Post Hoc Analysis Including Additional Adjustment for Age of the Pregnant Individual at Estimated Date of Delivery eTable 11. Chromosomal and Pregnancy Outcomes by Nuchal Translucency Measurement: Detailed Results eTable 12. Characteristics of Pregnancies With and Without Cytogenetic Testing [file jamanetwopen-e243689-s001.pdf]

Supplementary Online Content

Bellai-Dussault K, Dougan SD, Fell DB, et al. Ultrasonographic fetal nuchal translucency measurements and cytogenetic outcomes. *JAMA Netw Open*. 2024;7(3):e243689. doi:10.1001/jamanetworkopen.2024.3689

eTable 1. Description of Data Sources

eTable 2. Method of Ascertainment of Outcome

eFigure 1. Inclusion Flow

eFigure 2. Directed Acyclic Graph for Association Between Nuchal Translucency Measurement and Chromosomal Anomalies

eTable 3. Regression Models Including Pregnancies Identified Through Other Data Sources Beyond Multiple-Marker Screening

eTable 4. Regression Model With Varying Definition of Exposure by Nuchal Translucency Percentile

eTable 5. Sensitivity Analysis for Losses to Follow-Up

eTable 6. Regression Models Including All Pregnancies With Nuchal Translucency Measurements

eTable 7. Regression Models Including Only Pregnancies With an Estimated Date of Delivery From September 1, 2018, to March 31, 2021

eTable 8. Regression Models Excluding Pregnancies With an Estimated Date of Delivery From April 1, 2020, to March 31, 2021

eTable 9. Time of Cytogenetic Testing of Chromosomal Anomalies Identified by Nuchal Translucency Measurement

eTable 10. Post Hoc Analysis Including Additional Adjustment for Age of the Pregnant Individual at Estimated Date of Delivery

eTable 11. Chromosomal and Pregnancy Outcomes by Nuchal Translucency Measurement: Detailed Results

eTable 12. Characteristics of Pregnancies With and Without Cytogenetic Testing

This supplementary material has been provided by the authors to give readers additional information about their work.

© 2024 Bellai-Dussault K et al. *JAMA Network Open*.

eTable 1. Description of Data Sources

Better Outcomes Registry & Network (BORN) Ontario	BORN Ontario is a prescribed registry for the province of Ontario, Canada, designed to capture various encounters the pregnant individual and child have with the healthcare system.<br>The three laboratories that provide all multiple-marker screening in Ontario transfer records directly to BORN on a weekly basis, and include information on nuchal translucency measurements, biomarkers, and maternal clinical information.<br>There are two cell-free DNA screening laboratories established in Ontario that provide publicly-funded screening, as well as privately paid screening and both contribute to the registry.<br>All 9 cytogenetic laboratories in Ontario provide data to the registry through quarterly transfers and include all tests performed both prenatally as well as postnatally and on products of conception.<br>The registry also captures all hospital births and home births with a midwife in the province.<br>Additional information on BORN can be found in the 2021 publication by Murphy et al. as well as on the www.bornontario.ca website.
Canadian Institute for Health Information (CIHI)	The CIHI Discharge Abstract Database metadata (DAD) contains information from hospital discharges in Ontario, as well as other provinces and territories of Canada.<br>Data from CIHI was used in this study to supplement information on pregnancy outcomes including congenital anomalies. More information on CIHI can be found at https://www.cihi.ca/en/discharge-abstract-database-metadata-dad.<br>The following variables were supplemented using CIHI data:<br>- Pregnancy outcome<br>○ Fetal demise - ICD10 code : P95<br>○ Spontaneous abortion – ICD10 codes: O03;P01.8<br>○ Termination of Pregnancy – ICD10 codes: O04, O05, O06, P96.4<br>○ Congenital anomalies – ICD10 codes from Q00 to Q99

© 2024 Bellai-Dussault K et al. *JAMA Network Open*.

eTable 2. Method of Ascertainment of Outcome

Chromosomal anomaly	Traditional cytogenetic testing[a]	Microarray testing	cfDNA screening[b]	Newborn clinical exam
**Conditions formally screened by program**				
trisomy 21 and 18	Tested	Tested	NPV >99.9[c]	Diagnosis may be excluded [d]
**Secondary findings of the screening program**				
trisomy 13	Tested	Tested	NPV >99.9 [c]	Diagnosis may be excluded [d]
other autosomal aneuploidies	Tested	Tested	Not tested	Diagnosis may be excluded [d]
mosaic autosomal aneuploidies	Tested	Can be ascertained, unless low-level[e]	Not tested	Cannot exclude diagnosis [f]
triploidy	Tested	Tested	Tested in only one cfDNA screening platform[b]	Diagnosis may be excluded [d]
monosomy x	Tested	Tested	May be tested (opt-in or automatically tested depending on platform) [b]	Cannot exclude diagnosis [f]
mosaic monosomy x	Tested	Can be ascertained, unless low-level[e]	Not tested	Cannot exclude diagnosis [f]
other sex chromosome aneuploidies	Tested	Tested	May be tested (opt-in or automatically tested depending on platform) [b]	Cannot exclude diagnosis [f]
mosaic other sex chromosome aneuploidies	Tested	Can ascertain mosaicism, unless low- level[e]	Not tested	Cannot exclude diagnosis [f]
22q11.2 deletion, Cri-du-Chat, Angelman/Prader-Willi, 1p36 deletion syndromes	Not routinely ascertained	Tested	May be tested (opt in, private pay), NPV unclear[b]	Cannot exclude diagnosis [f]
Other copy number variants	Large deletions or duplications only [g]	Tested	Not tested	Cannot exclude diagnosis [f]

NPV, Negative predictive value

[a] *Traditional cytogenetic testing refers to rapid aneuploidy detection techniques (fluorescence in situ hybridization (FISH) or quantitative fluorescence-PCR (QFPCR)), or karyotype.*

[b] *Two cfDNA screening laboratories are established in Ontario, one using Single Nucleotide Polymorphism (SNP) Based Analysis while the other performs Chromosome Specific Sequencing through their Digital Analysis of Selected Regions (DANSR) technology. At the time of the study, one laboratory automatically screened for sex chromosome aneuploidies, while the other allowed to opt in. Only the SNP-based technology can screen for triploidy. The option to self-paid for microdeletion syndromes was also available.*

[c] *Program Report, Prenatal Screening Ontario. Published online December 2021. Accessed July 19, 2023. https://www.bornontario.ca/en/pso/resources/Remediated-PDFs-2020/PSO-Program-Report---FINAL-Dec-8-2021.pdf*

[d] *Recognizable clinical findings are expected at birth. Nussbaum R, McInnes R, Willard H. Thompson & Thompson Genetics in Medicine. Eight Edition. ELSEVIER; 2016.*

[e] *Armour CM, Dougan SD, Brock JA, et al. Practice guideline: joint CCMG-SOGC recommendations for the use of chromosomal microarray analysis for prenatal diagnosis and assessment of fetal loss in Canada. J Med Genet. 2018;55(4):215-221. Doi:10.1136/jmedgenet-2017-105013*

[f] *Clinical findings of this condition may not be easily and clearly identifiable at birth due to phenotype variability and syndromes that are less recognizable. Nussbaum R, McInnes R, Willard H. Thompson & Thompson Genetics in Medicine. Eight Edition. ELSEVIER; 2016.*

[g] *Microarray can detect smaller deletions and duplications not identifiable on karyotype: Wapner RJ, Martin CL, Levy B, et al. Chromosomal Microarray versus Karyotyping for Prenatal Diagnosis. N Engl J Med. 2012;367(23):2175-2184. doi:10.1056/NEJMoa1203382*

© 2024 Bellai-Dussault K et al. *JAMA Network Open*.

eFigure 1. Inclusion Flow

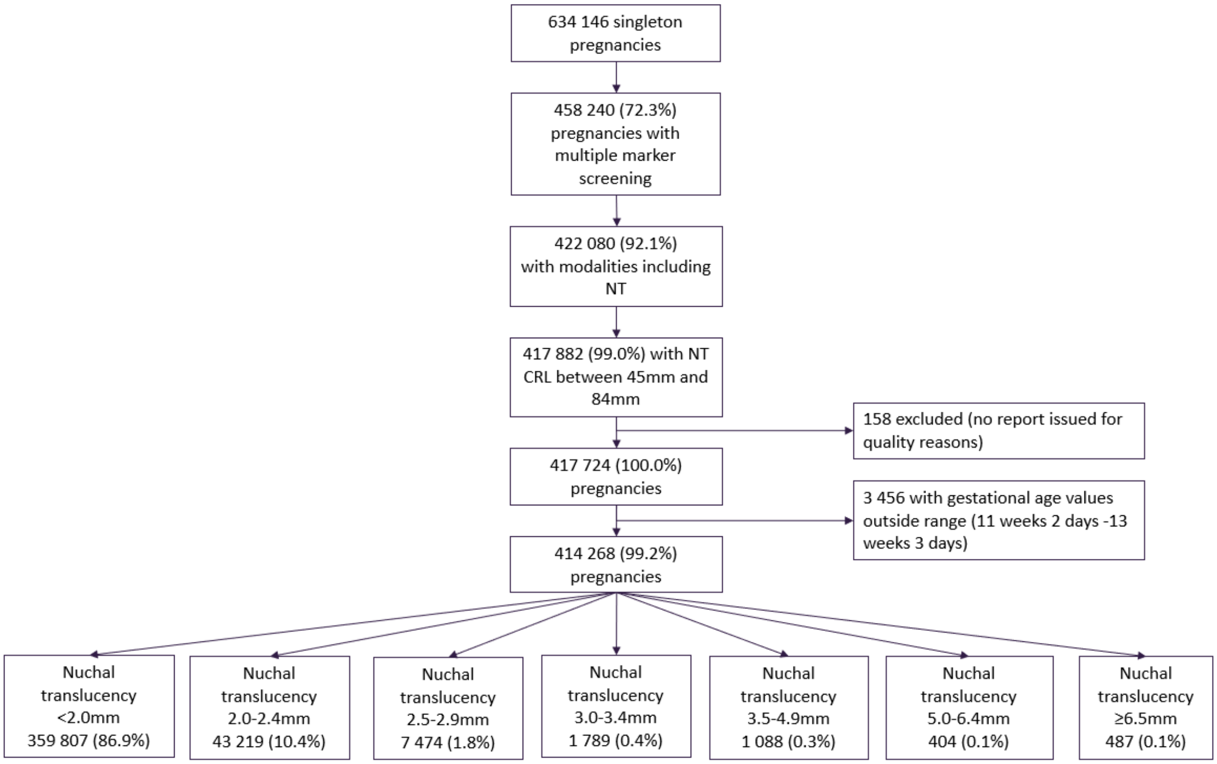

eFigure 2. Directed Acyclic Graph for Association Between Nuchal Translucency Measurement and Chromosomal Anomalies

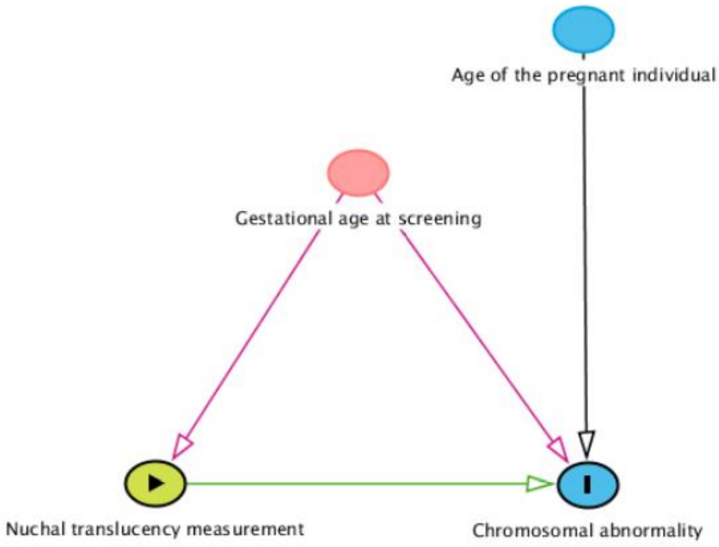

Note: Adjustment for factors that incidentally would affect the nuchal translucency measurement and would also be related to chromosomal anomalies were made to the regression models; only gestational age met this criterion. A post-hoc analysis also included adjustment for age of the pregnant individual and is presented in Supplementary Table 10.

© 2024 Bellai-Dussault K et al. *JAMA Network Open*.

eTable 3. Regression Models Including Pregnancies Identified Through Other Data Sources Beyond Multiple-Marker Screening

Nuchal translucency measurement	***Any chromosomal anomaly***			
	Risk Difference % (95% CI)		Risk Ratio (95% CI)	
< 2.0 mm	Ref.		Ref.	
2.0-<2.5 mm	0.55	(0.45-0.66)	1.97	(1.78-2.18)
2.5-<3.0 mm	2.24	(1.85-2.63)	4.92	(4.26-5.68)
3.0-<3.5 mm	9.95	(8.49-11.41)	18.42	(15.92-21.31)
≥ 3.5 mm	35.83	(33.85-37.82)	63.72	(59.39-68.37)

Outcome determined by cytogenetic testing, not having the outcome is determined by normal cytogenetic testing if performed, normal cfDNA screen if condition was tested and by live birth without clinical findings if condition can be clinically diagnosed.

Crude model is presented, as pregnancies with nuchal translucency measurements identified outside the multiple-marker screening data set did not have information on gestational age at screening.

Nuchal translucency measurements categories were collapsed, as the additional data sources only provided whether the nuchal translucency measurement was 3.0-<3.5 mm or ≥ 3.5 mm

eTable 4. Regression Model With Varying Definition of Exposure by Nuchal Translucency Percentile

Nuchal translucency percentile	***Any chromosomal anomaly***							
	Crude model				*Adjusted model**			
	Risk Difference % (95% CI)		Risk Ratio (95% CI)		Risk Difference % (95% CI)		Risk Ratio (95% CI)	
<90th percentile	Ref.		Ref.		Ref.		Ref.	
90-95th percentile	0.38	(0.26-0.50)	1.67	(1.46-1.91)	0.44	(0.32-0.57)	2.12	(1.84-2.43)
95-99th percentile	1.12	(0.94-1.29)	2.96	(2.64-3.30)	1.15	(0.98-1.32)	3.82	(3.39-4.29)
≥ 99th percentile	17.75	(16.69-18.82)	32.08	(29.80-34.53)	17.73	(16.66-18.79)	34.90	(32.43-37.57)

Outcome determined by cytogenetic testing, not having the outcome is determined by normal cytogenetic testing if performed, normal cfDNA screen if condition was tested and by live birth without clinical findings if condition can be clinically diagnosed.
**Model adjusted for gestational age at screening*

© 2024 Bellai-Dussault K et al. *JAMA Network Open*.

eTable 5. Sensitivity Analysis for Losses to Follow-Up

A

Nuchal translucency measurement	Number of pregnancies for which an outcome was recorded<br>n (%)	Prevalence of chromosomal anomaly among those for which an outcome was recorded<br>n (%)	Number of pregnancies for which NO outcome was recorded<br>n (%)	Number of pregnancies randomized to having a chromosomal anomaly. Assumption of half the prevalence of chromosomal anomalies compared to those for which an outcome is available.<br>n (%)	Number of pregnancies randomized to having a chromosomal anomaly. Assumption of twice the prevalence of chromosomal anomalies compared to those for which an outcome is available.<br>n (%)
< 2.0 mm	334858 (97.3)	1913 (0.6)	9281 (2.7)	26 (0.3)	103 (1.1)
2.0-<2.5 mm	40502 (97.3)	456 (1.1)	1132 (2.7)	6 (0.5)	25 (2.2)
2.5-<3.0 mm	7044 (97.5)	198 (2.7)	181 (2.5)	3 (1.4)	10 (5.5)
3.0-<3.5 mm	1701 (97.6)	179 (10.3)	42 (2.4)	3 (5.1)	9 (20.5)
3.5-<5.0 mm	1027 (96.4)	256 (24.0)	38 (3.6)	5 (12.0)	18 (48.1)
5.0-<6.5 mm	353 (90.1)	174 (44.4)	39 (9.9)	9 (22.2)	35 (88.8)
≥ 6.5 mm	425 (89.9)	256 (54.1)	48 (10.1)	13 (27.1)	48 (100.0)

Example

1913 (0.6%) of the 334 858 pregnancies with nuchal translucency measurement < 2.0 mm for which an outcome was recorded had a chromosomal anomaly. In this nuchal translucency category, 9281 (2.7%) pregnancies had no outcome recorded. In the sensitivity analyses, pregnancies lost to follow-up were randomized to having 1) half the prevalence of chromosomal anomalies (0.3% = 26 pregnancies randomly assigned to having a chromosomal anomaly) or 2) twice the prevalence of chromosomal anomalies (1.1% = 103 pregnancies randomly assigned to having a chromosomal anomaly).

© 2024 Bellai-Dussault K et al. *JAMA Network Open*.

B

Nuchal translucency measurement	***Main analysis***				***Sensitivity analysis****			
	Crude model		***Adjusted model****		***Adjusted model*** Pregnancies lost to follow-up assumed to have half the prevalence of chromosomal anomalies***		***Adjusted model*** Pregnancies lost to follow-up assumed to have twice the prevalence of chromosomal anomalies***	
	Risk Difference % (95% CI)	Risk Ratio (95% CI)	Risk Difference % (95% CI)	Risk Ratio (95% CI)	Risk Difference % (95% CI)	Risk Ratio (95% CI)	Risk Difference % (95% CI)	Risk Ratio (95% CI)
< 2.0 mm	Ref.	Ref.	Ref.	Ref.	Ref.	Ref.	Ref.	Ref.
2.0-<2.5 mm	0.55 (0.45-0.66)	1.97 (1.78-2.18)	0.61 (0.51-0.71)	2.39 (2.14-2.66)	0.60 (0.50-0.70)	2.38 (2.14-2.65)	0.63 (0.52-0.73)	2.37 (2.13-2.63)
2.5-<3.0 mm	2.24 (1.85-2.63)	4.92 (4.26-5.69)	2.26 (1.88-2.63)	5.93 (5.11-6.88)	2.22 (1.85-2.59)	5.91 (5.09-6.85)	2.31 (1.94-2.69)	5.87 (5.08-6.79)
3.0-<3.5 mm	9.95 (8.49-11.41)	18.42 (15.92-21.31)	9.94 (8.49-11.39)	20.33 (17.58-23.52)	9.81 (8.38-11.23)	20.30 (17.56-23.46)	10.19 (8.74-11.64)	20.21 (17.54-23.29)
3.5-<5.0 mm	24.36 (21.71-27.00)	43.63 (38.88-48.96)	24.31 (21.67-26.96)	42.94 (38.28-48.16)	23.90 (21.32-26.48)	42.82 (38.21-47.99)	25.10 (22.48-27.73)	43.27 (38.74-48.33)
5.0-<6.5 mm	48.72 (43.51-53.94)	86.28 (76.91-96.80)	48.68 (43.46-53.89)	80.85 (71.91-90.90)	46.07 (41.13-51.01)	76.76 (68.24-86.35)	52.68 (47.74-57.62)	84.55 (76.16-93.85)
≥ 6.5 mm	59.66 (54.99-64.33)	105.44 (96.40-115.32)	59.64 (54.97-64.31)	101.88 (92.79-111.88)	56.29 (51.81-60.76)	97.49 (88.76-107.10)	63.66 (59.33-68.00)	106.09 (97.57-115.35)

**Model adjusted for gestational age at screening*

***The sensitivity analyses randomly classified the losses to follow-up to having 1) half and 2) twice the prevalence of chromosomal anomalies compared to pregnancies in the same category of nuchal translucency measurement for which an outcome was recorded. (See supplementary Table 5A for details.)*

© 2024 Bellai-Dussault K et al. *JAMA Network Open*.

eTable 6. Regression Models Including All Pregnancies With Nuchal Translucency Measurements

Nuchal translucency measurement	***Any chromosomal anomaly***							
	Crude model				*Adjusted model**			
	Risk Difference % (95% CI)		Risk Ratio (95% CI)		Risk Difference % (95% CI)		Risk Ratio (95% CI)	
< 2.0 mm	Ref.		Ref.		Ref.		Ref.	
2.0-<2.5 mm	0.47	(0.32-0.63)	1.22	(1.15-1.30)	0.63	(0.47-0.79)	1.35	(1.26-1.44)
2.5-<3.0 mm	1.81	(1.37-2.25)	1.86	(1.66-2.08)	1.90	(1.47-2.34)	2.05	(1.82-2.30)
3.0-<3.5 mm	9.52	(8.04-11.01)	5.51	(4.84-6.28)	9.52	(8.04-10.99)	5.81	(5.10-6.61)
3.5-<5.0 mm	23.60	(21.00-26.21)	12.18	(10.98-13.51)	23.54	(20.94-26.13)	12.10	(10.92-13.42)
5.0-<6.5 mm	46.16	(41.28-51.03)	22.87	(20.62-25.36)	46.07	(41.20-50.94)	21.97	(19.80-24.37)
≥ 6.5 mm	55.95	(51.54-60.36)	27.51	(25.41-29.78)	55.91	(51.50-60.32)	27.01	(24.93-29.27)

Outcome determined by cytogenetic testing, not having the outcome is determined by normal cytogenetic testing if performed, normal cfDNA screen if condition was tested and by live birth without clinical findings if condition can be clinically diagnosed

If the outcome was not recorded, pregnancies were assumed to have the same risk of chromosomal anomalies as pregnancies with the same outcome, for which cytogenetic testing was performed. E.g., for the pregnancies that resulted in a pregnancy loss and had cytogenetic testing, 21.6% had a chromosomal anomaly, therefore 21.6% of pregnancies for which a pregnancy loss was recorded, but no cytogenetic testing was performed were randomized to have a chromosomal anomaly. The same process was followed for pregnancies for which cytogenetic outcomes were unavailable, but where a stillbirth, termination, live birth with clinical findings or loss to follow-up were recorded.

Pregnancies for which no outcome was recorded, but where a cfDNA screening result was high-risk, a positive predictive value of 90.0% was assumed and 90.0% of the pregnancies were randomized to have a chromosomal anomaly.

**Model adjusted for gestational age at screening.*

© 2024 Bellai-Dussault K et al. *JAMA Network Open*.

eTable 7. Regression Models Including Only Pregnancies With an Estimated Date of Delivery From September 1, 2018, to March 31, 2021

Nuchal translucency measurement	***Any chromosomal anomaly***							
	Crude model				*Adjusted model**			
	Risk Difference % (95% CI)		Risk Ratio (95% CI)		Risk Difference % (95% CI)		Risk Ratio (95% CI)	
< 2.0 mm	Ref.		Ref.		Ref.		Ref.	
2.0-<2.5 mm	0.52	(0.39-0.66)	1.98	(1.72-2.27)	0.58	(0.45-0.71)	2.41	(2.08-2.78)
2.5-<3.0 mm	2.20	(1.70-2.71)	5.12	(4.21-6.21)	2.22	(1.73-2.71)	6.19	(5.07-7.56)
3.0-<3.5 mm	10.56	(8.57-12.56)	20.74	(17.15-25.07)	10.56	(8.58-12.55)	22.99	(19.02-27.79)
3.5-<5.0 mm	24.85	(21.33-28.36)	47.42	(40.76-55.16)	24.80	(21.30-28.31)	46.94	(40.39-54.54)
5.0-<6.5 mm	50.91	(44.11-57.70)	96.10	(83.09-111.16)	50.87	(44.08-57.66)	89.97	(77.41-104.58)
≥ 6.5 mm	61.48	(55.56-67.40)	115.86	(103.43-129.77)	61.46	(55.53-67.38)	110.47	(97.98-124.55)

Indicates timeline with complete capture of microarray testing.Outcome determined by cytogenetic testing, not having the outcome is determined by normal cytogenetic testing if performed, normal cfDNA screen if condition was tested and by live birth without clinical findings if condition can be clinically diagnosed.

**Model adjusted for gestational age at screening*

eTable 8. Regression Models Excluding Pregnancies With an Estimated Date of Delivery From April 1, 2020, to March 31, 2021

Nuchal translucency measurement	***Any chromosomal anomaly***							
	Crude model				*Adjusted model**			
	Risk Difference % (95% CI)		Risk Ratio (95% CI)		Risk Difference % (95% CI)		Risk Ratio (95% CI)	
< 2.0 mm	Ref.		Ref.		Ref.		Ref.	
2.0-<2.5 mm	0.50	(0.38-0.62)	1.85	(1.64-2.08)	0.56	(0.44-0.68)	2.22	(1.96-2.51)
2.5-<3.0 mm	2.04	(1.61-2.46)	4.43	(3.74-5.25)	2.05	(1.64-2.47)	5.29	(4.44-6.30)
3.0-<3.5 mm	9.38	(7.78-10.99)	16.80	(14.20-19.88)	9.38	(7.78-10.97)	18.47	(15.61-21.85)
3.5-<5.0 mm	23.99	(20.99-27.00)	41.40	(36.28-47.25)	23.96	(20.95-26.96)	40.73	(35.70-46.47)
5.0-<6.5 mm	45.08	(38.95-51.20)	76.91	(66.65-88.74)	45.02	(38.90-51.15)	71.89	(62.22-83.05)
≥ 6.5 mm	59.28	(53.86-64.70)	100.83	(90.92-111.83)	59.26	(53.84-64.68)	97.93	(88.00-109.01)

Accounts for potential changes in practice during the COVID-19 pandemic. Outcome determined by cytogenetic testing, not having the outcome is determined by normal cytogenetic testing if performed, normal cfDNA screen if condition was tested and by live birth without clinical findings if condition can be clinically diagnosed

**Model adjusted for gestational age at screening*

© 2024 Bellai-Dussault K et al. *JAMA Network Open*.

eTable 9. Time of Cytogenetic Testing of Chromosomal Anomalies Identified by Nuchal Translucency Measurement

Time of cytogenetic testing for chromosomal anomalies identified	Nuchal translucency measurement (mm)							
	<2.0	2.0-<2.5	2.5-<3.0	3.0-<3.5	3.5-<5.0	5.0-<6.5	≥6.5	Total
Prenatal diagnosis, n (%)	490 (26.4)	204 (45.3)	119 (60.7)	134 (75.7)	196 (76.9)	135 (78.0)	165 (64.5)	1443 (42.9)
Postnatal cytogenetic testing, n (%)	1369 (73.6)	246 (54.7)	77 (39.3)	43 (24.3)	59 (23.1)	38 (22.0)	91 (35.5)	1923 (57.1)
Total	**1859**	**450**	**196**	**177**	**255**	**173**	**256**	**3366**

For 66 cytogenetic tests performed, the timing of the test was unknown

eTable 10. Post Hoc Analysis Including Additional Adjustment for Age of the Pregnant Individual at Estimated Date of Delivery

Nuchal translucency measurement	***Any chromosomal anomaly***									
	Crude model				*Model adjusted for gestational age at screening and age of the pregnant individual (continuous)**		*Model adjusted for gestational age at screening and age of the pregnant individual (categorical)***			
	Risk Difference % (95% CI)		Risk Ratio (95% CI)		Risk Ratio (95% CI)		Risk Difference % (95% CI)		Risk Ratio (95% CI)	
< 2.0 mm	Ref.		Ref.	Ref.	Ref.		Ref.		Ref.	Ref.
2.0-<2.5 mm	0.55	(0.45-0.66)	1.97	(1.78-2.18)	2.34	(2.10-2.61)	0.53	(0.43-0.63)	2.35	(2.11-2.61)
2.5-<3.0 mm	2.24	(1.85-2.63)	4.92	(4.26-5.69)	5.7	(4.92-6.61)	2.06	(1.70-2.42)	5.74	(4.96-6.66)
3.0-<3.5 mm	9.95	(8.49-11.41)	18.42	(15.92-21.31)	19.12	(16.57-22.06)	9.81	(8.37-11.25)	19.46	(16.85-22.47)
3.5-<5.0 mm	24.36	(21.71-27.00)	43.63	(38.88-48.96)	38.92	(34.80-43.53)	24.13	(21.49-26.76)	39.06	(34.86-43.76)
5.0-<6.5 mm	48.72	(43.51-53.94)	86.28	(76.91-96.80)	72.15	(64.30-80.96)	48.49	(43.28-53.69)	70.99	(63.09-79.87)
≥ 6.5 mm	59.66	(54.99-64.33)	105.44	(96.40-115.32)	87.96	(79.70-97.09)	59.47	(54.81-64.14)	87.23	(78.83-96.54)

**The model adjusted for age of the pregnant individuals using the continuous variable did not converge for the adjusted risk differences.*

***The age categories were defined as follows: (<25 years at estimated date of delivery, 25-<30 years , 30-<35 years, 35-<40 years, ≥40 years)*

© 2024 Bellai-Dussault K et al. *JAMA Network Open*.

eTable 11. Chromosomal and Pregnancy Outcomes by Nuchal Translucency Measurement: Detailed Results

Cytogenetic testing results	Pregnancies with nuchal translucency measurement and cytogenetic testing	Nuchal translucency measurement (mm)						
		<2.0	2.0-<2.5	2.5-<3.0	3.0-<3.5	3.5-<5.0	5.0-<6.5	≥6.5
**Total pregnancies with cytogenetic testing, No.**	**15,755**	**11,552**	**1,875**	**653**	**421**	**602**	**287**	**365**
**Unknown results, No. (%)**	**217 (1.4)**	**180 (1.6)**	**22 (1.2)**	**<6 (S)**	**<6 (S)**	**<6 (S)**	**<6 (S)**	**<6 (S)**
**No chromosomal anomaly identified, No. (%)**	**12106 (76.8)**	**9459 (81.9)**	**1397 (74.5)**	**450 (68.9)**	**241 (57.2)**	**341 (56.6)**	**112 (39.0)**	**106 (29.0)**
**Chromosomal anomalies identified results, No. (%)**	**3432 (21.8)**	**1913 (16.6)**	**456 (24.3)**	**198 (30.3)**	**179 (42.5)**	**256 (42.5)**	**174 (60.6)**	**256 (70.1)**
Trisomy 21	**1024 (29.8)**	299 (15.6)	180 (39.5)	127 (64.1)	117 (65.4)	151 (59.0)	74 (42.5)	76 (29.7)
Trisomy 18	**293 (8.5)**	110 (5.8)	20 (4.4)	11 (5.6)	13 (7.3)	32 (12.5)	50 (28.7)	57 (22.3)
Trisomy 13	**136 (4.0)**	39 (2.0)	23 (5.0)	10 (5.1)	10 (5.6)	25 (9.8)	16 (9.2)	13 (5.1)
Monosomy X	**114 (3.3)**	10 (0.5)	<6 (S)	0 (0.0)	<6 (S)	<6 (S)	11 (6.3)	83 (32.4)
Other sex chromosome aneuploidies	**123 (3.6)**	85 (4.4)	15 (3.3)	6 (3.0)	6 (3.4)	<6 (S)	<6 (S)	6 (2.3)
Triploidy	**61 (1.8)**	52 (2.7)	0 (0.0)	0 (0.0)	<6 (S)	<6 (S)	<6 (S)	<6 (S)
Mosaic or partial trisomies 21, 18 or 13	**59 (1.7)**	45 (2.4)	7 (1.5)	<6 (S)	<6 (S)	0 (0.0)	<6 (S)	0 (0.0)
Mosaic or partial sex chromosome aneuploidies	**154 (4.5)**	119 (6.2)	20 (4.4)	<6 (S)	<6 (S)	8 (3.1)	0 (0.0)	<6 (S)
22q11.2 microdeletion syndrome	**53 (1.5)**	40 (2.1)	<6 (S)	0 (0.0)	0 (0.0)	<6 (S)	0 (0.0)	0 (0.0)
Cri-du-Chat syndrome	**<6 (S)**	0 (0.0)	0 (0.0)	<6 (S)	0 (0.0)	0 (0.0)	0 (0.0)	0 (0.0)
Angelman or Prader-Willi syndrome	**12 (0.3)**	12 (0.6)	0 (0.0)	0 (0.0)	0 (0.0)	0 (0.0)	0 (0.0)	0 (0.0)
1p36 deletion	**<6 (S)**	<6 (S)	0 (0.0)	0 (0.0)	0 (0.0)	0 (0.0)	0 (0.0)	0 (0.0)

© 2024 Bellai-Dussault K et al. *JAMA Network Open*.

Other autosomal aneuploidies	**<6 (S)**	<6 (S)	0 (0.0)	<6 (S)	<6 (S)	<6 (S)	0 (0.0)	0 (0.0)
Other mosaic autosomal aneuploidies	**12 (0.3)**	<6 (S)	<6 (S)	<6 (S)	<6 (S)	<6 (S)	0 (0.0)	<6 (S)
Other chromosomal anomaly	**1384 (40.3)**	1095 (57.2)	175 (38.4)	37 (18.7)	22 (12.3)	27 (10.5)	14 (8.0)	14 (5.5)
Pathogenic finding	***421***	*311*	*59*	*12*	*10*	*13*	*10*	*6*
Likely Pathogenic finding	***91***	*76*	*8*	*<6 (S)*	*<6 (S)*	*<6 (S)*	*<6 (S)*	*0*
Variant of Uncertain Significance	***717***	*598*	*85*	*21*	*<6 (S)*	*<6 (S)*	*<6 (S)*	*<6 (S)*
No interpretation available	***155***	*108*	*23*	*<6 (S)*	*6*	*7*	*<6 (S)*	*6*
**Results from cfDNA screening for those without cytogenetic testing**	**Pregnancies with nuchal translucency measurement and cfDNA (but no cytogenetic testing)**	**Nuchal translucency measurement (mm)**						
		**<2.0**	**2.0-<2.5**	**2.5-<3.0**	**3.0-<3.5**	**3.5-<5.0**	**5.0-<6.5**	**≥6.5**
**Total pregnancies with cfDNA screening, No.**	**38,754**	**30,551**	**5,310**	**1,701**	**667**	**398**	**69**	**58**
No results, No. (%)	**495 (1.3)**	404 (1.3)	58 (1.1)	14 (0.8)	<6 (S)	8 (2.0)	<6 (S)	<6 (S)
Low risk, No. (%)	**38041 (98.2)**	30071 (98.4)	5234 (98.6)	1674 (98.4)	645 (96.7)	354 (88.9)	44 (63.8)	19 (32.8)
High risk T21, No. (%)	**103 (0.3)**	18 (0.1)	15 (0.3)	12 (0.7)	16 (2.4)	21 (5.3)	9 (13.0)	12 (20.7)
High risk T18	**38 (0.1)**	13 (0.0)	0 (0.0)	<6 (S)	<6 (S)	7 (1.8)	7 (10.1)	9 (15.5)
High risk T13, No. (%)	**15 (0.0)**	6 (0.0)	0 (0.0)	0 (0.0)	0 (0.0)	<6 (S)	<6 (S)	<6 (S)
High risk monosomy X, No. (%)	**20 (0.1)**	6 (0.0)	<6 (S)	0 (0.0)	0 (0.0)	<6 (S)	0 (0.0)	11 (19.0)
High risk other SCA, No. (%)	**18 (0.0)**	16 (0.1)	0 (0.0)	0 (0.0)	0 (0.0)	<6 (S)	<6 (S)	0 (0.0)
High risk triploidy, No. (%)	**22 (0.1)**	16 (0.1)	<6 (S)	0 (0.0)	0 (0.0)	<6 (S)	<6 (S)	0 (0.0)
High risk microdeletion, No. (%)	**<6 (S)**	<6 (S)	0 (0.0)	0 (0.0)	<6 (S)	0 (0.0)	0 (0.0)	0 (0.0)

© 2024 Bellai-Dussault K et al. *JAMA Network Open*.

eTable 12. Characteristics of Pregnancies With and Without Cytogenetic Testing

**Characteristic**	**Pregnancies with nuchal translucency measurement**	**Nuchal translucency measurement**					
		**<2.0 mm**		**2.0-<3.5 mm**		**≥3.5 mm**	
		Did not have cytogenetic testing	Had cytogenetic testing	Did not have cytogenetic testing	Had cytogenetic testing	Did not have cytogenetic testing	Had cytogenetic testing
**Total**	**414,268**	**348,255**	**11,552**	**49,533**	**2,949**	**725**	**1,254**
**Maternal age at EDD, years** Mean (SD)	**31.5 (4.7)**	31.4 (4.7)	32.5 (5.1)	31.6 (4.8)	33.4 (5.2)	32.2 (5.4)	33.4 (5.1)
**Gestational age at screening, days** Mean (SD)	**87.8 (3.3)**	87.5 (3.3)	87.3 (3.3)	90 (2.7)	88.8 (3.1)	87.3 (3.7)	86.9 (3.5)
**Crown Rump Length, mm** Mean (SD)	**62.5 (8.3)**	61.7 (8.1)	61.2 (8.1)	68.6 (7.6)	65.4 (8.3)	61.4 (9.0)	60.4 (8.4)
**Maternal weight, kg** Mean (SD)	**68 (17.0)**	67.8 (16.9)	69.3 (18.5)	68.5 (17.3)	68.5 (18.2)	68.5 (17.4)	66.6 (15.6)
**Parity**, No. (%)							
Nulliparous	**183587 (46.2)**	157256 (46.8)	4738 (44.7)	20201 (42.4)	930 (37.7)	222 (45.5)	240 (38.0)
Primiparous	**143190 (36.0)**	119978 (35.7)	3683 (34.8)	18194 (38.2)	919 (37.2)	171 (35.0)	245 (38.8)
Multiparous	**70967 (17.8)**	58681 (17.5)	2167 (20.5)	9258 (19.4)	619 (25.1)	95 (19.5)	147 (23.3)
Missing	***16,524***	*12,340*	*964*	*1,880*	*481**	*237**	*622**
**Conception,** No. (%)							
Spontaneous conception	**374873 (96.0)**	316437 (96.0)	10013 (94.6)	44468 (95.9)	2497 (95.3)	546 (95.6)	912 (97.3)
IVF	**12025 (3.1)**	9970 (3.0)	459 (4.3)	1465 (3.2)	93 (3.5)	17 (3.0)	21 (2.2)
Other ART	**3659 (0.9)**	3075 (0.9)	113 (1.1)	429 (0.9)	30 (1.1)	8 (1.4)	<6 (S)
Missing	***23,711***	*18,773*	*967*	*3,171*	*329**	*154**	*317**
**Smoking status**, No. (%)							
Nonsmoker	**358781 (91.8)**	302150 (92.0)	9888 (91.4)	42473 (90.5)	2533 (91.0)	629 (92.2)	1108 (93.9)
Smoker	**32117 (8.2)**	26334 (8.0)	930 (8.6)	4477 (9.5)	251 (9.0)	53 (7.8)	72 (6.1)
Missing	**23,370**	19,771	734	2,583	165	43	74

**Missingness >10.0%*

SD, standard deviation; IVF, in vitro fertilization; ART, Assisted reproductive technology

© 2024 Bellai-Dussault K et al. *JAMA Network Open*.
