# Supplementary material for: Ultrasonographic Fetal Nuchal Translucency Measurements and Cytogenetic Outcomes
Source: JAMA Netw Open. 2024 Mar 26;7(3):e243689. doi: 10.1001/jamanetworkopen.2024.3689 (PMC10966411; doi:10.1001/jamanetworkopen.2024.3689)
Supplement: Supplement 2. — Data Sharing Statement [file jamanetwopen-e243689-s002.pdf]

Data Sharing Statement

Bellai-Dussault. Ultrasonographic Fetal Nuchal Translucency Measurements and Cytogenetic Outcomes. *JAMA Netw Open*. Published March 26, 2024.
doi:10.1001/jamanetworkopen.2024.3689

Data
Data available: No

Additional Information
Explanation for why data not available: This study used secondary data routinely collected through the BORN registry and all analyses were performed in the secure environment of the registry. Only aggregate results were released for the purposes of this publication to comply with the privacy requirements of the registry.
